# Supplementary material for: Identification of photoperiod-induced specific miRNAs in the adrenal glands of Sunite sheep (Ovis aries)
Source: Front Vet Sci. 2022 Jul 22;9:888207. doi: 10.3389/fvets.2022.888207 (PMC9354845; doi:10.3389/fvets.2022.888207)
Supplement: Supplementary file 1 [file Data_Sheet_1.ZIP › Supplementary file/miRDeep2 file.zip!/miRDeep2 file/Know_Structure/SPLP42A3/oar-let-7b.pdf]

miRBase precursor : oar-let-7b
Total read count : 112269
oar-let-7b read count : 111824
remaining reads : 445

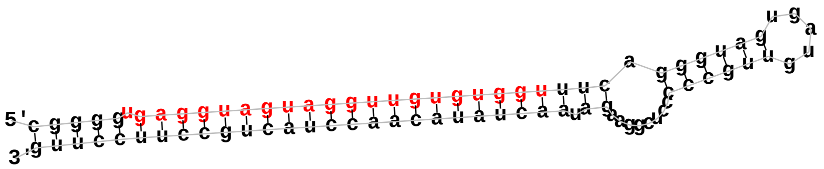

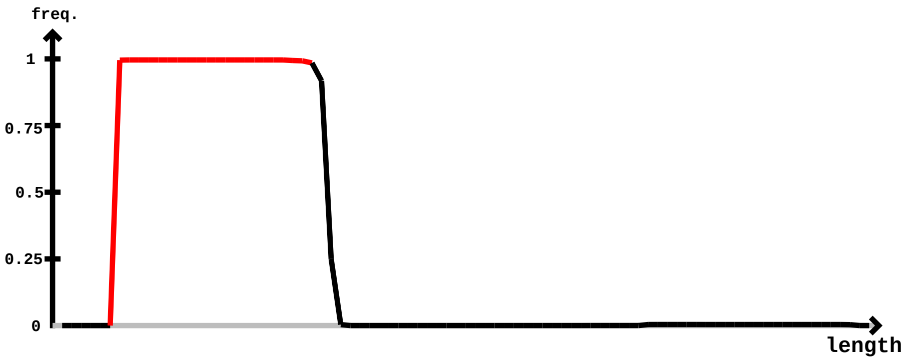


```
      oar-let-7b
5'- cggggugagguaguagguuguguggu uucaggguagugauguugcccccucggaagauaacuauacaaccuacugccuuccuug   -3'    exp
    (((((.(((((((((((((((((((((((.((((((.....))))))(....)..)).))))))))))))))))))))))))))   reads      mm         sample
    .....ugaCguaguagguugug..............................................................   1          1          seq
    .....ugagguaguagguugug..............................................................   19         0          seq
    .....ugagguaguaAguugugu.............................................................   1          1          seq
    .....ugagguaguagguugugu.............................................................   223        0          seq
    .....ugagguaguagguugugug............................................................   141        0          seq
    .....ugagguaguagguugugugg...........................................................   881        0          seq
    .....ugagguaguagguuguguggu..........................................................   7517       0          seq
    .....ugagguaguagguugugugguu.........................................................   74906      0          seq
    .....ugagguaguagguugugugguuu........................................................   27637      0          seq
    .....ugagguaguagguugugugguuuc.......................................................   413        0          seq
    ......gagguaguagguuguguggu..........................................................   3          0          seq
    ......gagguaguagguugugugguu.........................................................   60         0          seq
    ......gagguaguagguugugugguuu........................................................   20         0          seq
    .......agguaguagguugugugguuu........................................................   1          0          seq
    .........guaguagguugugugguuu........................................................   1          0          seq
    ...........................ucaggguagugauguugcccccucggaagaua.........................   1          0          seq
    ............................................cccccucggaagauaacuauac..................   1          0          seq
    .....................................................aagauaacuauacaaccuacugc........   1          0          seq
    ......................................................agauaacuauacaaccuacugc........   3          0          seq
    .........................................................uaacuauacaaccuacugccuu.....   1          0          seq
    ............................................................cuauacaaccuacugccuuc....   4          0          seq
    ............................................................cuauacaaccuacugccuucc...   49         0          seq
    ............................................................cuauacaaccuacugccuuccu..   366        0          seq
    ............................................................cuauacaaccuacugccuuccuu.   15         0          seq
    .............................................................uauacaaccuacugccuuccu..   4          0          seq
```
